# Prognostic role of metformin intake in diabetic patients with colorectal cancer: An updated qualitative evidence of cohort studies

**DOI:** 10.18632/oncotarget.14688

**Published:** 2017-01-17

**Authors:** Lili Du, Mingli Wang, Yingying Kang, Bo Li, Min Guo, Zhifeng Cheng, Changlong Bi

**Affiliations:** ^1^ Department of Endocrinology, The Fourth Affiliated Hospital of Harbin Medical University, Harbin, China

**Keywords:** metformin, anti-diabetic drug, colorectal cancer, prognosis, diabetes mellitus

## Abstract

Several observational studies have shown that metformin can modify the risk and survival of colorectal cancer (CRC) in patients with diabetes mellitus, although the magnitude of this relationship has not been determined. We conducted an updated systematic review and meta-analysis to analyze the association between metformin and CRC mortality and searched relevant databases up to July 2016. The primary outcome was overall survival (OS). Secondary outcomes were cancer-specific survival (CS) and disease-free survival (DFS). Summary hazard ratios (HRs) were calculated using a random-effects model. Seventeen studies enrolling 269,417 participants were eligible for inclusion. Comparing with non-metformin users in diabetic CRC patients, the summary HRs for OS in metformin users were 0.69 (95% CI, 0.61-0.77). Subgroup analyses stratified by the study characteristics and sensitivity analysis by the trim-and-fill method (adjusted HR 0.77, 95% CI, 0.67-0.87) confirmed the robustness of the results. However, significant OS benefit was noted in patients with stage II and III disease. Five studies reported the CRC prognosis for CS and three for DFS; metformin intake was significantly associated with patient CS (HR 0.75, 95% CI, 0.59-0.94), but not DFS (HR 0.38, 95% CI, 0.13-1.17). Our findings suggest that metformin intake is associated with improved survival outcomes in terms of OS and CS in CRC patients with diabetes, particular for OS in stage II and stage III patients. Further studies should be conducted to determine CRC survival between metformin use and patient specific clinical and molecular profiles.

## INTRODUCTION

As a commonly used oral anti-hyperglycaemic agent for type 2 diabetes mellitus (DM), metformin can improve insulin sensitivity by increasing peripheral glucose intake and utilization [1]. It has also been indicated that insulin might play an important role in tumorigenesis. Numerous epidemiologic studies have reported that metformin is associated with decreased risk of cancer incidence [2–7]. Metformin is supposed to inhibit cellular growth and proliferation by lowering insulin levels through the phosphoinositide 3-kinase (PI3K)/protein kinase B (Akt)/ mammalian target of rapamycin (mTOR) pathway pathways [8, 9]. Recent published study has also shown that metformin exhibits an anti-apoptotic effect on podocytes under high glucose conditions through activation of adenosine monophosphate-activated protein kinase (AMPK) and inhibition of mTOR signaling [10]. More and more researches have focused on the potential role of metformin as a combined drug for patients with cancer. Metformin intake has been reported to be associated with reduced risk of colorectal cancer (CRC) as well as improved survival in CRC patients [11–18], such as pathologic complete response in locally advanced rectal cancer treated with chemoradiotherapy [19]. Several meta-analyses have also examined the association between metformin and CRC survival [20, 21]. However, these systematic reviwews are quite preliminary with limited number of studies included and low statistical power to draw a definite conclusion. Moreover, the findings of previous meta-analyses were difficult to interprete due to heterogeneity in patient/population selection, metformin exposure, outcome measures, and study design. During the last two years, several related large cohorts were studied and the results were published [13, 17, 22]. Therefore, there is an urgent need to conduct an updated meta-analysis to systematically renew the evidence about the association between metformin intake and survival outcomes in CRC patient with DM.

## RESULTS

### Search and selection of studies

**Figure 1:**
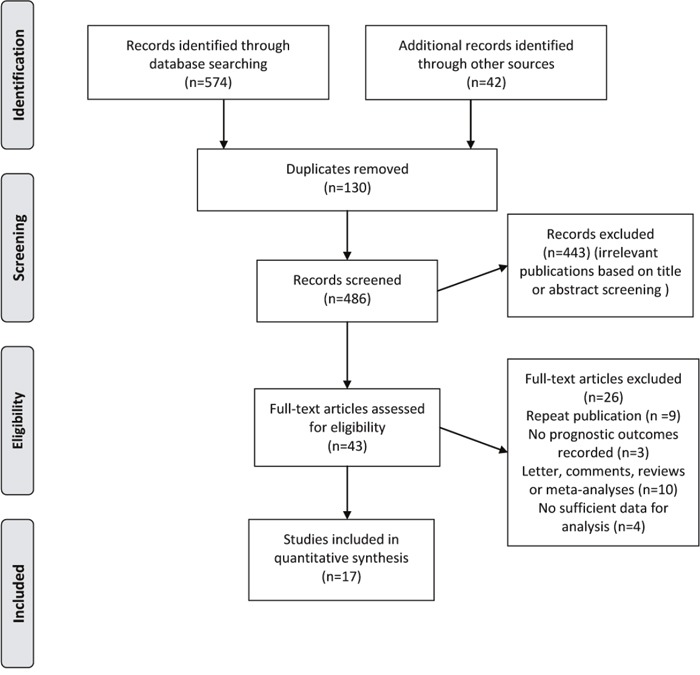
Flow diagram of selection of studies investigating effect of metformin intake on survival in patients with colorectal cancer

Literature search generated a total of 616 citations, of which 43 appeared relevant through title/abstract selection and were retrieved for further full text evaluation (Figure 1). Finally, 17 studies met all eligibility criteria and were included in the meta-analysis [11–17, 19, 22–30] (Figure 1 and Supplementary Table 1).

### Study characteristics

Seventeen observational studies with a total of 269,417 individuals were involved in the study. Eight took place in USA and Canada (North America), six in Asia and three in Europe. Ten studies recruited participants from multiple centers, while seven recruited exclusively from single centers. The median number of sample size per study was 3816 (range, 482-111673). Nine of the included studies were population-based cohort studies and eight were hospital-based cohort studies. Two studies enrolled patients with rectal cancer only, one study with only colon cancer, while the other studies included patients with both colon and rectal cancer. Thirteen of the seventeen studies examined patients with CRC of all stages (I-IV), while two studies enrolled only stage III and stage IV patients, respectively, and another reported I-III stage patients. Based on NOS criteria, out of nine possible points, five studies received nine points, three received eight points, three received seven points, two received six points, three received five point, and two received four points (Table 1).

**Table 1:** Characteristics of the included studies on survival outcomes of metformin use and colorectal cancer survival

Authors (Ref.)	Study design	Country	No. of hospitals involved	Metformin user/non-user	Sample size	Tumor site	Disease stage	Source of data	Median follow-up duration	Score of methodological assessment	Survival endpoints	Adjusted variables
BansalM CR et al, 2011	Retrospective cohort study	USA	Multiple	141/430	1078	CRC	II-IV	VA cancer registry database (VISN16)	NA	4	OS	Chemotherapy modalities, such as single agent or multi-agent
Garrett, et al, 2012	Retrospective cohort study	USA	Single	208/216	4758	CRC	I-IV	MD Anderson Cancer Institute	NA	7	OS	Age, sex, race, BMI, aspirin usage, and initial stage of disease
Lee GE, et al, 2012	Retrospective cohort study	Singapore	Single	219/125	1455	CRC	II-III	Singapore hospital based study.	78 months	6	OS, RFS	T stage, N stage and patients' age
Lee JH, et al, 2012	Retrospective cohort study	Korea	Single	258/337	6108	CRC	I-IV	Korean hospital based study.	41 months (range, 1-119 )	8	CS	Age at diagnosis, sex, stage of cancer, BMI, diabetes duration, smoking status, HbA1c level, use of aspirin and use of insulin, sulfonylurea and thiazolidinediones.
Cossor FI et al, 2013	Large, prospective cohort study	USA	Multiple	84/212	2066	CRC	I-IV	WHI study	4.1 years (range, 3 day-14.4 years).	9	OS CS	Age and tumor stage
Skinner HD et al, 2013	Retrospective cohort study	USA	Single	20/40	482	RC	I-IV	MD Anderson Cancer Institute	NR	7	OS DFS	NR
Spillane S et al, 2013	National, prospective cohort	Ireland	Multiple	207/315	3816	CRC	I-III	National Cancer Registry Ireland study	≥4 years	9	OS CS	Age, tumor stage, tumor grade, year of diagnosis, comorbidity score, aspirin use, exposure to non-metformin ADDs, socioeconomic status, and radiation therapy
Paulus JK et al, 2014	Retrospective cohort study	USA	Multiple	2038/2136	21352	CRC	I-IV	US Veterans Health Administration study	NR	5	OS	Age, race, stage, body mass index, HbA1c, comorbidity index, and cancer treatment.
Al Omari A et al, 2015	Retrospective cohort study	Amman	Single	56/215	1898	CRC	I-IV	King Hussein Cancer Center Study	NR	5	OS	Age, gender, body mass index, and stage
He WZ et al, 2015	Retrospective cohort study	China	Single	45/91	2542	CRC	IV	Sun Yat-Sen University Cancer Center study	26 months	5	OS PFS	NR
Paul Singh P et al, 2015	Prospective cohort study	USA	Multiple	115/152	1958	CC	III	NCCTG N0147 (Alliance) Study	NR	4	DFS OS RFS	KRAS, BRAF mutation status, tumor site, T/N stage, gender, and age
Xu H et al, 2015	Retrospective cohort study	USA	Multiple	389/290	111673	CRC	I-IV	Vanderbilt cohort and Mayo Clinic cohort	NR	8	CS	Age, sex, race, BMI, tobacco use, insulin, cancer type, and non-cancer Charlson index
Zanders MM et al, 2015	Population-based cohort study	The Netherlands	Multiple	666/377	8725	CRC	I-IV	ECR-PHARMO cohort	3.4 years (s.d.±3.0)	9	OS	Sex, age at CRC diagnosis, calender year of CRC diagnosis, type of CRC, stage at CRC diagnosis and administration of surgery, radiotherapy and/or chemotherapy, use of other diabetes medication, statins and aspirin
Fransgaard T et al, 2015	Population-based cohort study	Denmark	Multiple	1962/1429	30493	CRC	I-IV	Danish Colorectal Group's National Clinical Database	NR	7	OS	Age, sex, ASA score, BMI, blood transfusions, smoking, alcohol consumption, elective or emergency surgery, type of cancer, T stage, lymph node status, and distant metastasis, diabetic complications
Ki YJ et al, 2016	Population-based retrospective cohort study	Korea	Multiple	3649/ 809	58124	RC	I-IV	Korea Center Cancer Registry study	NR	8	OS, CS	Sex, age, SEER stage, Charlson's comorbidity index score, Preoperative chemotherapy, Adjuvant chemotherapy
Mc Menamin UC et al, 2016	Retrospective cohort study	Northern Ireland	Multiple	675/552	11595	CRC	I-IV	NCDR, CPRD and ONS death registrations.	4 years ranged from 6 months to 14 years.	9	OS, CS	Gender, year of diagnosis, age at diagnosis, deprivation, site, surgery within 6 months, radiotherapy within 6 months, chemotherapy within 6 months, comorbidities. other anti-diabetic medication usage,and other medication usage
Ramjeesingh R et al, 2016	Retrospective cohort study	Canada	Single	133/144	1304	CRC	I-IV	The Cancer Centre of Southeastern Ontario	NR	6	OS	Age, sex, comorbidities, diabetes treatments, BMI, smoking history, alcohol history, family history of crc, location of cancer, stage at diagnosis, differentiation

Abbreviations: ADDs, anti-diabetic drugs; ASA, American Society of Anesthesiologists; BMI, body mass index;BRAF, BRAF mutation; CRC, colorectal cancer; CS, cancer-specific survival; DFS, disease-free survival; KRAS, KRAS mutation; N, N stage; NR, not reported; OS, overall survival; RFS, recurrence-free survival; SEER, the Surveillance, Epidemiology, and End Results; T, T stage.

Important baseline characteristics of the included studies in the meta-analysis are presented in Table 1. Seventeen studies investigated the association between metformin use and OS for CRC patients [11–17, 19, 22–30], whereas five [13, 14, 16, 22, 24] and three [19, 29, 31] studies reported the CS and DFS, respectively. Three and four studies contained survival data of exclusively colon and rectal cancer, respectively. However, the majority of the included studies involved both colon and rectal cancer patients. Patients with a mixture of stage I-IV disease was investigated in fourteen studies and four other studies included stage II, IV or I-III cancer patients. The median follow-up period ranged from 26 months to more than four years from the available follow-up information. Age, gender, body mass index, and stage were four most commonly investigated variables that were adjusted for in Cox's proportional-hazard model evaluation of the association between metformin intake and CRC mortality.

### Metformin intake and CRC prognosis

**Figure 2:**
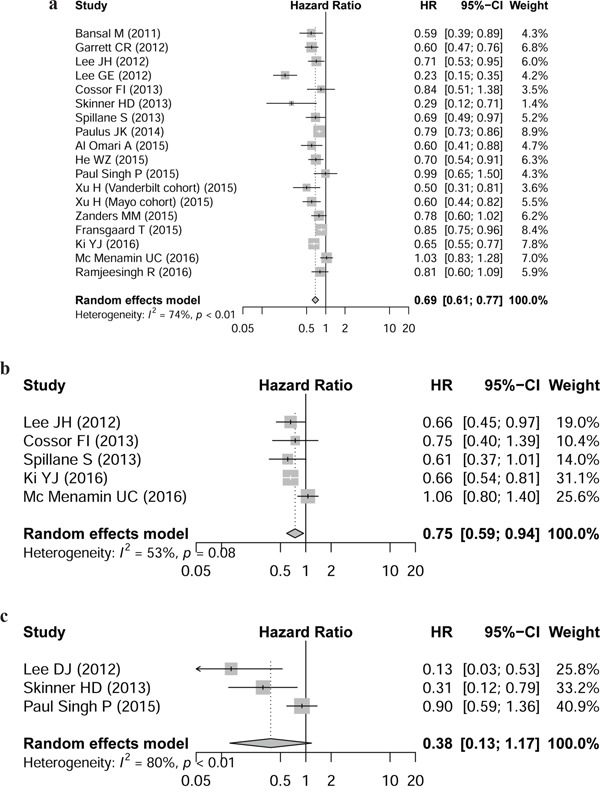
Hazard ratio for association between *metformin intake* and a. overall survival, b. cancer-specific survival, c. disease-free survival Forest plots of 17 cohorts. Weights are from random effects analysis. Abbreviations: CI, confidence interval; HR, hazard ratio; W (random): Weights (random effects model).

Meta-analysis based on all observational studies assessing CRC OS in patients with DM indicated that compared with non-use, metformin use was significant associated with decreased OS in patients with DM (HR 0.69, 95 % CI 0.61-0.77, Figure 2), with considerable heterogeneity across studies (I^2^=73.5%). The analysis of studies when involving prospective cohorts showed similar results with low heterogeneity (HR 0.83, 95% CI 0.75-0.92, I^2^=0%).

Five studies reported the CRC prognosis for CS and three for DFS; metformin intake was significantly associated with patient CS (HR 0.75, 95% CI, 0.59-0.94), but not DFS (HR 0.38, 95% CI, 0.13-1.17).

### Subgroup analyses

The effect of metformin use on patient OS appeared to be smaller among studies investigating only colon cancers (pooled HR 0.83, 95% CI 0.67-1.03, three studies, I^2^=0%) compared with studies including only rectal cancers (HR 0.73, 95% CI 0.58-0.91, four studies, I^2^=41.4%, Table 2). However, we did not note statistically significant difference between this subgroup (*P* for interaction =0.40).

**Table 2:** Subgroup analyses of the associations between metformin use and overall survival

Comparison variables	Overall survival		
	N (I^2^ statistics %, *P*_het_)	HR 95% CI	*P*_interaction_
Total	17 (73.5, <0.001)	0.69 [0.61; 0.77]	NA
Study design			0.004
Prospectively	5 (0, 0.704)	0.83 [0.75; 0.92]	
Retrospectively	12 (78.8, <0.001)	0.63 [0.54; 0.74]	
Patient origin			0.005
Population-based	9 (49.4, 0.045)	0.79 [0.72; 0.88]	
Hospital-based	8 (72.8, <0.001)	0.56 [0.45; 0.70]	
Research country			0.066
North America	8 (54.2, 0.026)	0.69 [0.59; 0.80]	
Europe	3 (0, 0.048)	0.82 [0.74; 0.91]	
Asia	6 (87.4, <0.001)	0.62 [0.45; 0.84]	
Centers involved			0.037
Single	7 (79.1, <0.001)	0.56 [0.42; 0.74]	
Multiple	10 (54.9, 0.014)	0.76 [0.69; 0.85]	
Sample size			0.923
<5000	9 (25.2, 0.220)	0.69 [0.60; 0.79]	
≥5000	8 (84.2, <0.001)	0.68 [0.57; 0.80]	
NOS score			0.102
<7	6 (80.7, <0.001)	0.76 [0.69; 0.84]	
≥7	11 (11.2, 0.344)	0.64 [0.54; 0.77]	
Tumor location			0.40
Colon	3 (0, 0.392)	0.83 [0.67; 1.03]	
Rectum	4 (41.4, 0.172)		0.73 [0.58; 0.91]
Tumor stage			0.367
Stage I	2 (0, 0.936)	0.98 [0.47; 2.02]	
Stage II	2 (0, 0.921)	0.73 [0.57; 0.93]	
Stage III	3 (0, 0.882)	0.58 [0.37; 0.92]	
Stage IV	6 (56.6, 0.042)	0.90 [0.67; 1.20]	
Stage I/II/III	3 (72.4, 0.027)		0.82 [0.61; 1.10]

Abbreviations: CI, confidence interval; het, heterogeneity; HR, hazard ratio; N, number of studies; NA, not available.

The prognostic effect of metformin intake on patient OS was larger in studies that limited their analyses in retrospective studies (HR 0.63, 95% CI 0.54-0.74, twelve studies, I^2^=78.8%) compared with the estimates from studies that limited their analyses in prospective studies (HR 0.83, 95% CI 0.75-0.92, five studies, I^2^=0%). Significant subgroup difference was observed (*P* for interaction =0.004).

The prognostic effect of metformin intake was also noted significantly large in the subgroup of patient origin from hospital-based studies (HR 0.56, 95% CI 0.45-0.70, I^2^=72.8%; Table 2), compared with that from population-based studies (HR 0.79, 95% CI 0.72-0.88, I^2^=49.4%, *P* for interaction =0.005). Another possible interaction was noted in subgroup of different number of centers involved for OS (*P* for interaction =0.037).

Results of other subgroup analyses are presented in Table 2, which showed that the prognostic effect remained stable for patients with different research region, sample size and NOS score. When the analyses were limited to individual CRC tumor stage, we noted that statistical prognistic effect was noted only in stage II and stage III patients, but not in stage I and stage IV patients.

### Sensitivity analyses and publication bias

**Figure 3:**
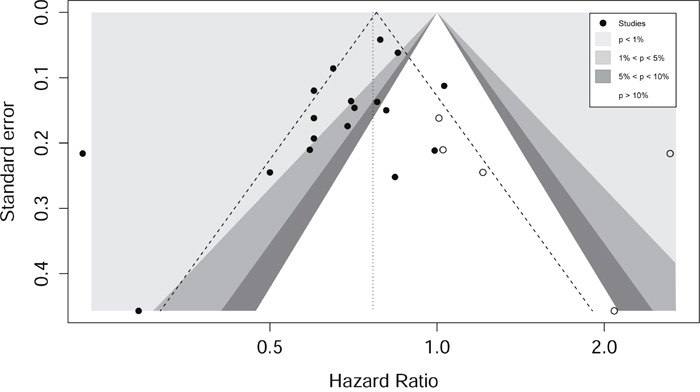
Contour enhanced funnel plot for meta-analysis of the association between metformin intake and overall survival The plots indicates that most studies were in the significant areas where P<0.01 and where 0.01<P<0.05, while few studies were in the non-significant area (the blank area). Circles refer to included studies, and four missing studies (white circles) were filled.

For OS subset, the funnelplot indicated asymmetry and the presence of publication bias (Figure 3). The hollow circles showed that three missing studies that lay in the non-significant regions of the plot, suggesting that asymmetry was attributed partly to publication bias, which was further confirmed with Egger's linear regression test (*P*=0.037). The adjusted random effects pooled HR of 0.77 (95% CI, 0.67-0.87) calculated using the trim-and-fill method remained constant with the original analysis. We did not explore the publication bias for CS or DFS due to the limited number of studies involved.

## DISCUSSION

This meta-analysis based on patient survival data from observational studies comparing metformin use and non-use in patients with CRC revealed that metformin intake was associated with better outcomes in terms of OS and CS.

The significant association between metformin intake and OS of patients was also noted in most baseline subsets, further confirming the prognostic role of metformin in CRC. However, the significant associations were exhibited in only rectal cancer patients and stage II and stage III patients, probably due to the limited number of studies involved.

A significant heterogeneity (I^2^= 73.5%) of the analysis was observed in this systematic review for OS. In sensitivity analysis, exclusion of each of the studies in turn did not largely alter the significance of the pooled estimate. Subgroup analysis showed that the heterogeneity significantly reduced for most investigated baseline characteristics (Table 2), indicating that the source of inter-study heterogeneity may partly attribute to these variables. Moreover, subgroup analysis indicated that metformin intake was significantly correlated with improved OS for CRC patients in stage II (HR 0.73; 95% CI 0.57-0.93), stage III tumors (HR 0.58 95% CI 0.37-0.92) and rectal cancer (HR 0.73; 95% CI 0.58-0.91), while not in other stages or in colon cancer. As the influential factors and characteristics of CRC in different locations and stages might differ because of diverse molecular profiles and epigenetic background of tumors, the prognosis of CRC might vary among different tumor locations and stages. However, though it did not reach statistical significance for some subsets, the trends all indicated that patients with metformin intake had favorable OS, probably due to the limited sample size or statistical power involved. More prospective and multicenter studies might further clarify its real prognostic role in specific cancer profiles.

The finding that stage II/III CRC patients who used metformin had more survial benefits than stage I/IV patients, which was consistent with the findings of some previously published studies [12, 14]. We proposed that some of the stage II and almost all of the stage III patients would receive adjuvant chemotherapy, which played an important role in the clinical outcomes of these patients. Therefore, we suggested that metformin had a potential synergic effect with other anti-cancer agents and might be a candidate drug as an additional therapy to adjuvant chemotherapy in stage II/III CRC (locally advanced CRC). However, limited sample size were included and caution should be taken when when interpreting these results.

Several explanations for the potential association between metformin intake and CRC patient survival were proposed. It was reported that metformin enhanced tumor necrosis factor-related apoptosis-inducing ligand-induced apoptosis through the degradation of myeloid cell leukemia-1 (MCL-1) by the proteasome machinery [32]. Metformin has also an inhibitory effect on cell proliferation mediated by suppression of mTOR and insulin-like growth factor 1 (IGF1)/AKT pathways [33]. In addition, Langer et al. has found that metformin can increase cell viability which was associated with reduced apoptosis. This factor is also associated with increased AMPK and Akt or reduced mTOR in a concentration-dependent manner, thus mediating pro-survival effects after apoptosis-inducing high glucose stimulation [10]. Still, previous research has also suggested that the biologic effects of metformin are mediated through reexpression of miRNAs and decreased expression of cancer stem cell-specific genes, indicating that metformin is beneficial for overcoming therapeutic resistance of certain cancer cells [34].

For subset of OS, significant between-study heterogeneity was noted (I^2^=73.5%, *P*_heterogeneity_ <0.001). The result of sensitivity analyses showed that exclusion any one of the study did not greatly change the pooled estimate. The results of the trim-and-fill method and subgroup analyses stratified by the major clinical variables had the same trend with the main analyses, indicating the robustness of our findings which were not influenced by publication bias. However, still caution should be taken when interpreting the findings, as publication bias is unavoidable and the statistical analyses for publication bias are not perfectly comprehensive.

Three previous meta-analyses have examined metformin intake for predicting CRC patient survival [20, 21, 35]. The first two meta-analyses by Mei et al and Zhang et al found that CRC patients with type 2 DM who took metformin had longer OS and CS based on six and five cohort studies, respectively, which was reported again by He et al in 2016. However, due to the limited number of studies and small sample size, no sufficient statistical power of these two systmatic reviews has been added, so updated evidence is urgently needed for further clarify this associations.

This meta-analysis is the most comprehensive summary to provide robust statistical evidence for the substantial prognostic impact of metformin intake in CRC patients with type 2 DM. Despite the fact that several recently published studies showed a positive association between metformin and survival in CRC patients [11–15, 17, 19], the small sample size (ranging from 67 to 616) could not provide sufficient statistical power to draw a definite conclusion. These inconsistent findings could result from several factors. Firstly, the included studies had different study designs. Eight studies applied a convenience sample from single centers while other nine studies selected cases from multiple institutions. Nine studies used population-based cohorts while eight used hospital-based cohorts in epidemiologic settings. These variations would inevitably invlolve in selection bias. Secondly, the included studies covered a wide range of sample sizes from 106 to 111673. We known that smaller studies in a meta-analysis could show larger treatment effects, leading to inaccurate estimates of effect sizes for the investigated association, and publication bias are very likely to occur. Asymmetry was clearly noted in the funnel plot (Figure 3). However, we used the trim-and-fill model for adjustment, and the result agreed with the primary analysis, suggesting the significant association between metformin intake and CRC patient survival. Thirdly, the included studies involved various disease characteristics including tumor stage, location and other molecular features, which were other sources of heterogeneity. Our subgroup analyses have shown that metformin intake was significantly associated with patient OS only in stage II, stage III patients and rectal cancer patients, but not in stage I, stage IV patients or colon cancer patients. Nevertheless, these effects should be interpreted with caution due to limited studies in each studies and lack of sufficient statitical power. The findings of these subgroups should be further validated from more large-scale cohorts.

Limitations of this systematic review should not be ignored. Firstly, most of the studies did not have the information of the molecular date concerning KRAS, BRAF, PIK3CA mutation or microsatellite status, patient information of each tumor stage, chemotherapy or radiotherapy patient received, so sensitivity analyses could not be performed based on these variables. Secondly, as the included ones were all study-level studies, we could not abstract more detailed information of each individual, for example, duration and doses of metformin use, the stage of diabetes, concentration of blood glucose, and the amount of metformin intake for each people, etc, thus some of the subgroup analyses we were interested in could not be performed. Thirdly, the heterogeneity of statistical analyses lies in the difference in the adjustment variables among studies, which could have affected the accuracy and precision of the combined estimates. Multivariate Cox proportional hazards models were used in almost all of the studies except two [13, 19] that did not directly report survival estimates. We calculated the survival estimates using the method provided by Parmar et al. [36], by which uncertain bias, such as immortal time bias, for pooled estimates could have occured. Recently, it has been criticized that the effect of metformin on cancer survival might be exaggerated due to immortal time bias. However, some study showed that the presence of immortal time bias would not change the direction of metformin's effect on lung cancer [37]. We did sensitivity analysis by excluding studies that did not used multivariate Cox proportional hazards models and the direction of the result remained unchanged. Fourthly, we did not search unpublished gray literature, which could have led to publication bias.

Our study has several strengths. First, we thoroughly searched the four major databases without language or publication date limits, minimizing the risk of missing publications which might cause publication bias. Second, this meta-analysis is the largest and most comprehensive, including the biggest sample size of over 269,000 individuals, providing the solidest evidence for this topic currently. Third, we had done thorough stratified analysis based on some of the major study characteristics, such as the study design, participant features, follow-up period and NOS scale for study quality, and the findings were generally constant independent of some of the study characteristics. Fourth, we selected and cross-checked the identified studies, developed and abstracted the data, rated the study quality at least by two to three independent reviewers, minimizing the selection bias to the greatest extent and more objectively performing the systematic review.

In summary, our findings suggest that meformin intake is associated with improved survival outcomes in terms of OS and CS in CRC patients with DM, particular for OS in stage II and stage III patients. Further studies should be conducted to determine CRC survival between metformin use and patient specific clinical and molecular profiles.

## MATERIALS AND METHODS

### Search strategy

A literature search of the major databases including PubMed, Embase, the Cochrane Library Central Register of Controlled Trials and American Society of Clinical Oncology (ASCO) databases from inception to July 2016 was performed for all relevant studies investigating an association between metformin use and CRC prognostic outcomes. Supplementary Table 2-5 present the detailed search strategies using the following free-text words combined with Medical Subject Headings (MeSH) or EMTREE terms: “colorect*/colon*/rectum/rectal”, “cancer*/tumor*/tumour*/carcinom*/neoplas* /adenocarcinoma* /malignan*”, “metformin/biguanide*”, and “prognos*/survival/recurren*/mortality/predict*/outcome*/death”. Manual reference search was also conducted for recent citations in some of the high impact journals such as Annals of Oncology, Diseases of the Colon & Rectum and the Journal of the National Cancer Institute. We also scrutinized for additional citations from the references of primary selected studies, reviews or meta-analyses which were not identified through database search. We did not apply date or language restrictions to our search strategy.

### Study selection and inclusion criteria

Three reviewers (LD, MW and YK) independently evaluated all the titles and abstracts identified through the primary literature search, then selected and identified all the potentially relevant citations retrieved for full text reviews to assess for eligibility. Any disagreements were resolved by consensus or by a senior reviewer (CB or ZC) if necessary.

Observational studies were considered eligible for inclusion if: (1) studies published with original data in peer-review journals or published in style of abstracts without language restrictions; (2) studies at least reporting one of the survival outcomes, such as overall survival (OS), cancer-specific survival (CS) and disease-free survival (DFS); and (3) studies investigating the impact of metformin intake in CRC patients and providing prognostic data with a hazard ratio (HR) estimate and its 95% confidence interval (CI) comparing survival outcomes of metformin users with that of in non-users. We excluded the following studies if: (1) studies containing no prognostic data; (2) studies having no sufficient information concerning prognostic parameters for analysis; and (3) study type including letters, comments or reviews without original data.

When duplicate publication suspected, we used the study with the largest sample size or contacted the corresponding authors and asked for clarification via e-mail if possible.

A comprehensive data abstraction form for each study containing the following baseline characteristics were extracted: the first author name, study design, research country, number of hospitals involved in the research, number of patients taking metformin and not taking metformin, the sample size of the full cohort, tumor site, disease stage, source of data, median follow-up duration, survival outcomes with their estimates (HRs with corresponding 95% CIs) and adjusted variables.

We used the 9-star Newcastle-Ottawa Scale (NOS) [38] to evaluate study quality, which was a validated tool for the assessment of the methodological quality of non-randomized studies. Three domains including selection of the participants, comparability of the participants and outcomes were allocated to 9 points. We proposed a score of more than 6 as high risk of bias.

### Statistical analysis

Data analysis was synthesized using R software version 3.1.2. and Stata® version 12.0 (StataCorp LP, College Station, Texas, USA). We expressed time-to event data as HRs with 95% CIs. Between-study heterogeneity was tested using I^2^ statistic defined as an I^2^ value >50% indicating substantial heterogeneity. As substantial clinical, methodological, or statistical heterogeneity among included studies, we pooled the data using a random-effects model for the meta-analysis or using the method reported by Parmar et al [36]. We investigated the source of heterogeneity through subgroup analyses by examining potential influencial variables that could explain some of the heterogeneity. Differences between subgroups were evaluated using the test of subgroup differences described by Deeks et al [39]. Publication bias was assessed by visual inspection of the funnel plot symmetry combined with Begg's regression or Egger's linear regression method [40, 41], with a *P* value less than 0.05 indicating statistically significant. We also tested the possible impact of publication bias through Duval's non-parametric trim-and-fill method [42].

## SUPPLEMENTARY TABLES


