# Supplementary material for: Prognostic role of metformin intake in diabetic patients with colorectal cancer: An updated qualitative evidence of cohort studies
Source: Oncotarget. 2017 Jan 17;8(16):26448–59. doi: 10.18632/oncotarget.14688 (PMC5432271; doi:10.18632/oncotarget.14688)
Supplement: Supplementary file 1 [file oncotarget-08-26448-s001.pdf]

# Prognostic role of metformin intake in diabetic patients with colorectal cancer: An updated qualitative evidence of cohort studies

## SUPPLEMENTARY DATA

**Supplementary Table 1: Selection procedure of included and excluded studies**

| | Reference list no. |
|---|---|
| Studies included in the meta-analysis (n = 17) | 1-17 |
| Studies excluded from meta-analysis, and reasons are listed below (n = 26) | 18-43 |
| Reason for exclusion | |
| Publication with cases included in a selected study (n =9) | 18-26 |
| No prognostic outcomes recorded (n =3) | 27-29 |
| Letters, comments, reviews or meta-analyses (n =10) | 30-39 |
| No sufficient data for analysis (n =4) | 40-43 |

## Search strategy

Search included: Pubmed, Embase, the Cochrane Library Central Register of Controlled Trials and American Society of Clinical Oncology (ASCO): till 26 July, 2016.

## REFERENCES

1. Bansal M, Siegel E and Govindarajan R. The effect of metformin (M) on overall survival (OS) of patients (Pts) with colorectal cancer (CRC) treated with chemotherapy (CTX). Journal of Clinical Oncology. 2011; 29.
2. Garrett CR, Hassabo HM, Bhadkamkar NA, Wen S, Baladandayuthapani V, Kee BK, Eng C and Hassan MM. Survival advantage observed with the use of metformin in patients with type II diabetes and colorectal cancer. British journal of cancer. 2012; 106:1374-1378.
3. Lee GE, Aung T, Lim KH, Tan WS, Tai WMD, Suhaimi NAB, Tan MH and Tan IB. Examining the effects of metformin on survival outcome in stage II/III colorectal cancer patients with diabetes mellitus. Journal of Clinical Oncology. 2012; 30.
4. Lee JH, Kim TI, Jeon SM, Hong SP, Cheon JH and Kim WH. The effects of metformin on the survival of colorectal cancer patients with diabetes mellitus. International journal of cancer. 2012; 131:752-759.
5. Cossor FI, Adams-Campbell LL, Chlebowski RT, Gunter MJ, Johnson K, Martell RE, McTiernan A, Simon MS, Rohan T, Wallace RB and Paulus JK. Diabetes, metformin use, and colorectal cancer survival in postmenopausal women. Cancer epidemiology. 2013; 37:742-749.
6. Skinner HD, Crane CH, Garrett CR, Eng C, Chang GJ, Skibber JM, Rodriguez-Bigas MA, Kelly P, Sandulache VC, Delclos ME, Krishnan S and Das P. Metformin use and improved response to therapy in rectal cancer. Cancer medicine. 2013; 2:99-107.
7. Spillane S, Bennett K, Sharp L and Barron TI. A cohort study of metformin exposure and survival in patients with stage I-III colorectal cancer. Cancer epidemiology, biomarkers & prevention. 2013; 22:1364-1373.
8. Paulus JK, Cossor FI, Williams CD, Martell RE and Kelley MJ. Metformin (M), diabetes (DM), and colorectal cancer (CRC) survival among U.S. Veterans. Journal of Clinical Oncology. 2014; 32.
9. Al Omari A, Abdelkaleq H, Al-Hussaini M, Turfa R, Awad N, Hassan M and Garrett CR. The metformin effect on survival in Middle Eastern patients (pts) with type II diabetes (DM) and colorectal cancer (CRC). Journal of Clinical Oncology. 2015; 33.
10. He WZ and Xia LP. Impact of metformin on survival in patients with type II diabetes and metastatic colorectal cancer. Journal of Clinical Oncology. 2015; 33.
11. Paul Singh P, Shi Q, Foster NR, Grothey A, Nair S, Chan E, Shields AF, Goldberg RM, Gill S, Kahlenberg MS, Sinicrope FA, Sargent DJ and Alberts SR. Relationship between metformin use and recurrence and survival in patients (pts) with resected stage III colon cancer (CC) receiving adjuvant chemotherapy: Results from NCCTG N0147 (Alliance). Journal of Clinical Oncology. 2015; 33.
12. Xu H, Aldrich MC, Chen Q, Liu H, Peterson NB, Dai Q, Levy M, Shah A, Han X, Ruan X, Jiang M, Li Y, Julien JS, Warner J, Friedman C, Roden DM, et al. Validating drug repurposing signals using electronic health records: a case study of metformin associated with reduced cancer mortality. Journal of the American Medical Informatics Association. 2015; 22:179-191.
13. Zanders MM, van Herk-Sukel MP, Vissers PA, Herings RM, Haak HR and van de Poll-Franse LV. Are metformin, statin and aspirin use still associated with overall mortality among colorectal cancer patients with diabetes if adjusted for one another? British journal of cancer. 2015; 113:403-410.
14. Fransgaard T, Thygesen LC and Gogenur I. Metformin Increases Overall Survival in Patients with Diabetes Undergoing Surgery for Colorectal Cancer. Annals of surgical oncology. 2016; 23:1569-1575.
15. Ki YJ, Kim HJ, Kim MS, Park CM, Ko MJ, Seo YS, Moon SM and Choi JA. Association Between Metformin Use and Survival In Non-Metastatic Rectal Cancer Treated with a Curative Resection: A Nationwide Population Study. Cancer research and treatment. 2016.
16. Mc Menamin UC, Murray LJ, Hughes CM and Cardwell CR. Metformin use and survival after colorectal cancer: A population-based cohort study. International journal of cancer. 2016; 138:369-379.
17. Ramjeesingh R, Orr C, Bricks CS, Hopman WM and Hammad N. A retrospective study on the role of diabetes and metformin in colorectal cancer disease survival. Current oncology (Toronto, Ont). 2016; 23:e116-122.
18. Lee JH, Kim TI, Jeon SM, Hong SP, Cheon JH and Kim WH. The effects of metformin on survival after diagnosis of colorectal cancer in diabetic patients. Gastroenterology. 2011; 140:S681.
19. Cossor FI, Adams-Campbell LL, Chlebowski RT, Gunter MJ, Johnson K, Martell RE, McTiernan A, Simon MS, Rohan T, Wallace RB and Paulus J. Diabetes, metformin use, and colorectal cancer survival in women: A retrospective cohort study. Journal of Clinical Oncology. 2012; 30.
20. Lee DJ, Kim B, Lee JH, Park SJ, Hong SP, Cheon JH, Kim TI and Kim WH. The effect of metformin on responses to chemotherapy and survival in stage IV colorectal cancer with diabetes. [Aricle in Korean].The Korean journal of gastroenterology. 2012; 60:355-361.
21. Spillane S, Bennett K, Sharp L and Barron TI. Effects of metformin and sulfonylureas on overall and colorectal

cancer-specific mortality. Journal of Clinical Oncology. 2012; 30.

22. Zanders M, Van Herk-Sukel M, Haak H, Herings R, Lemmens V and Van De Poll-Franse L. Statin use as a moderator of metformin effect on overall survival in colorectal cancer patients with diabetes. European Journal of Cancer. 2013; 49:S308-S309.
23. Omari AA, Abdelkaleq H, Al-Hussaini M, Turfa R, Awad N, Al Mutar SS, Hassabo HM, Sahin IH, Hassan M and Garrett CR. Retrospective evaluation of Middle Eastern patients (pts) with type II diabetes mellitus (DM) and colorectal cancer (CRC): Validation of metformin effect on overall survival (OS). Journal of Clinical Oncology. 2014; 32.
24. Fransgaard T, Thygesen L and Gögenür I. Metformin use among patients with diabetes improves overall survival after surgery for colorectal cancer. Diseases of the colon and rectum. 2015; 58:e360-e361.
25. Park JW, Kim SB, Lee JH, Lee HJ, Park SJ, Hong SP, Cheon JH, Kim WH and Kim TI. Gender-dependent difference in the effect of metformin on colorectal cancer-specific mortality of diabetic colorectal cancer patients. Journal of Gastroenterology and Hepatology (Australia). 2015; 30:99-100.
26. Park C, Choi J, Kim H, Ko M, Kim Y, Kang S and Jo A. Oncologic outcomes of metformin in patients with non-metastatic colon cancer and type 2 diabetes in South Korea using nationwide database. Value in Health. 2016; 19:A142.
27. Spillane S, Bennett K, Sharp L and Barron TI. Metformin exposure and disseminated disease in patients with colorectal cancer. Cancer epidemiology. 2014; 38:79-84.
28. Oh B, Kim H, Lee W, Cho Y, Park Y, Yun S and Huh J. Metformin enhances the response to radiotherapy in rectal cancer. Diseases of the colon and rectum. 2015; 58:e195.
29. Oh BY, Park YA, Huh JW, Cho YB, Yun SH, Lee WY, Park HC, Choi DH, Park YS and Kim HC. Metformin enhances the response to radiotherapy in diabetic patients with rectal cancer. Journal of cancer research and clinical oncology. 2016; 142:1377-1385.
30. Gronich N and Rennert G. Beyond aspirin - Cancer prevention with statins, metformin and bisphosphonates. Nature Reviews Clinical Oncology. 2013; 10:625-642.
31. Zhang P, Li H, Tan X, Chen L and Wang S. Association of metformin use with cancer incidence and mortality: a meta-analysis. Cancer epidemiology. 2013; 37:207-218.
32. Lega IC, Shah PS, Margel D, Beyene J, Rochon PA and Lipscombe LL. The effect of metformin on mortality following cancer among patients with diabetes. Cancer epidemiology, biomarkers & prevention. 2014; 23:1974-1984.
33. Mei ZB, Zhang ZJ, Liu CY, Liu Y, Cui A, Liang ZL, Wang GH and Cui L. Survival benefits of metformin for colorectal cancer patients with diabetes: a systematic review and meta-analysis. PloS one. 2014; 9:e91818.
34. Miranda VC, Barroso-Sousa R, Glasberg J and Riechelmann RP. Exploring the role of metformin in anticancer treatments: A systematic review. Drugs of Today. 2014; 50:623-640.
35. Singh PP, Singh S, Gonsalves WI and Grothey A. Association of metformin with reduced mortality in patients with colorectal cancer: A systematic review and meta-analysis of observational studies. Journal of Clinical Oncology. 2014; 32.
36. Zhang ZJ and Li S. The prognostic value of metformin for cancer patients with concurrent diabetes: a systematic review and meta-analysis. Diabetes, obesity & metabolism. 2014; 16:707-710.
37. Sehdev A and O'Neil BH. The Role of Aspirin, Vitamin D, Exercise, Diet, Statins, and Metformin in the Prevention and Treatment of Colorectal Cancer. Current treatment options in oncology. 2015; 16:43.
38. Anisimov VN. Metformin for Prevention and Treatment of Colon Cancer: A Reappraisal of Experimental and Clinical Data. Current drug targets. 2016; 17:439-446.
39. He XK, Su TT, Si JM and Sun LM. Metformin Is Associated With Slightly Reduced Risk of Colorectal Cancer and Moderate Survival Benefits in Diabetes Mellitus: A Meta-Analysis. Medicine. 2016; 95:e2749.
40. Hassabo HM, Hassan M, George B, Wen S, Baladandayuthapani V, Kopetz S, Fogelman DR, Kee BK, Eng C and Garrett CR. Survival advantage associated with metformin usage in patients with colorectal cancer (CRC) and type II noninsulin-dependent diabetes (NIDDM). Journal of Clinical Oncology. 2011; 29.
41. Ramjeesingh R, Orr C, Richardson L and Hammad N. A retrospective study on the role of diabetes and metformin in colorectal cancer disease progression. Journal of Clinical Oncology. 2013; 31.
42. Sahin IH, Hassabo HM, Shen Y, Kee BK, Hassan M and Garrett CR. Validation of the survival benefit from metformin use in patients with type 2 diabetes and colorectal cancer. Journal of Clinical Oncology. 2013; 31.
43. Miranda VC, Faria LD, Braghiroli MIFM, Jacobs M, Sabbaga J, Hoff PM and Riechelmann RP. A phase II trial of metformin and fluorouracil (MetFU) for patients (pts) with metastatic colorectal cancer (mCRC) refractory to standard treatment. Journal of Clinical Oncology. 2014; 32.

**Supplementary Table 2: Search strategy for Pubmed (Publication date to 2016/07/26)**

1. “ Biguanides”[Mesh]
2. “ Metformin”[Mesh]
3. 1 OR 2
4. (metformin OR Biguanides) [Title/Abstract]
5. 3 OR 4
6. “Colorectal Neoplasms”[Mesh]
7. “Colonic Neoplasms”[Mesh]
8. “Rectal Neoplasms”[Mesh]
9. (((colorect*[Title/Abstract]) OR colon*[Title/Abstract]) OR rectum[Title/Abstract]) OR rectal[Title/Abstract]
10. ((((((cancer*[Title/Abstract]) OR tumor*[Title/Abstract]) OR tumour*[Title/Abstract]) OR carcinom*[Title/Abstract]) OR neoplas*[Title/Abstract]) OR adenocarcinoma*[Title/Abstract]) OR malignan*[Title/Abstract]
11. 9 AND 10
12. 6 OR 7 OR 8 OR 11
13. “Mortality”[Mesh]
14. “Survival”[Mesh]
15. “Prognosis”[Mesh]
16. ((((((prognos*[Title/Abstract]) OR survival[Title/Abstract]) OR recurren*[Title/Abstract]) OR mortality[Title/Abstract]) OR predict*[Title/Abstract]) OR outcome*[Title/Abstract]) OR death[Title/Abstract]
17. 13 OR 14 OR 15 OR 16
18. 5 AND 12 AND 17

**Supplementary Table 3: Search strategy for Embase (Publication date to 2016/07/26)**

1.‘metformin’/exp
2. ‘biguanide derivative’/exp
3. 1 OR 2
4. (metformin OR biguanides):ab,ti
5. 3 OR 4
6. ‘colorectal cancer’/exp
7. ‘colon cancer’/exp
8. ‘rectum cancer’/exp
9. ((colorect* OR colon* OR rectum OR rectal) and (cancer* OR tumor* OR tumour* OR carcinom* OR neoplas* OR adenocarcinoma* OR malignan*)):ab,ti
10. 6 OR 7 OR 8 OR 9
11. ‘mortality’/exp
12. ‘survival’/exp
13. ‘cancer prognosis’/exp
14. (prognos* OR survival OR recurren* OR mortality OR predict* OR outcome* OR death):ab,ti
15. 11 OR 12 OR 13 OR 14
16. 5 AND 10 AND 15

**Supplementary Table 4: Search strategy for the Cochrane Library Central Register of Controlled Trials (Publication date to 2016/07/26)**

1. MeSH descriptor: [Metformin] explode all trees
2. MeSH descriptor: [Biguanides] explode all trees
3. 1 OR 2
4. (metformin or Biguanides):ti,ab,kw
5. 3 OR 4
6. MeSH descriptor: [Colorectal Neoplasms] explode all trees
7. MeSH descriptor: [Colonic Neoplasms] explode all trees
8. MeSH descriptor: [Rectal Neoplasms] explode all trees
9. (colorect* or colon* or rectum or rectal) and (cancer* or tumor* or tumour* or carcinom* or neoplas* or adenocarcinoma* or malignan*):ti,ab,kw
10. 6 OR 7 OR 8 OR 9
11. MeSH descriptor: [Mortality] explode all trees
12. MeSH descriptor: [Survival] explode all trees
13. MeSH descriptor: [Prognosis] explode all trees
14. prognos* or survival or recurren* or mortality or predict* or outcome* or death:ti,ab,kw
15. 11 OR 12 OR 13 OR 14
16. 5 AND 10 AND 15

**Supplementary Table 5: Search strategy for American Society of Clinical Oncology (ASCO) database (http://jco.ascopubs.org/search)**

| *The ASCO database (1983 to July 26 2016) were searched with the following search strategy* |
|---|
| 1. metformin OR Biguanides |
| 2. colorect* OR colon* OR rectum OR rectal |
| 3. cancer* OR tumor* OR tumour* OR carcinom* OR neoplas* OR adenocarcinoma* OR malignan |
| 4. 2 AND 3 |
| 5. prognos* OR survival OR recurren* OR mortality OR predict* OR outcome* OR death |
| 6. 1 AND 4 AND 5 |
